# Analysis of Pre-Analytic Factors Affecting the Success of Clinical Next-Generation Sequencing of Solid Organ Malignancies

**DOI:** 10.3390/cancers7030859

**Published:** 2015-08-28

**Authors:** Hui Chen, Rajyalakshmi Luthra, Rashmi S. Goswami, Rajesh R. Singh, Sinchita Roy-Chowdhuri

**Affiliations:** 1 Department of Pathology, The University of Texas MD Anderson Cancer Center, 1515 Holcombe Blvd, Houston, TX 77030, USA; E-Mails: hchen7@mdanderson.org (H.C.); sroy2@mdanderson.org (S.R.-C.); 2 Department of Hematopathology, The University of Texas MD Anderson Cancer Center, 1515 Holcombe Blvd, Houston, TX 77030, USA; E-Mails: goswami.rashmi@gmail.com (R.S.G.); rsingh@mdanderson.org (R.R.S.)

**Keywords:** next-generation sequencing, targeted hotspot mutation analysis, pre-analytic factors, tissue qualification

## Abstract

Application of next-generation sequencing (NGS) technology to routine clinical practice has enabled characterization of personalized cancer genomes to identify patients likely to have a response to targeted therapy. The proper selection of tumor sample for downstream NGS based mutational analysis is critical to generate accurate results and to guide therapeutic intervention. However, multiple pre-analytic factors come into play in determining the success of NGS testing. In this review, we discuss pre-analytic requirements for AmpliSeq PCR-based sequencing using Ion Torrent Personal Genome Machine (PGM) (Life Technologies), a NGS sequencing platform that is often used by clinical laboratories for sequencing solid tumors because of its low input DNA requirement from formalin fixed and paraffin embedded tissue. The success of NGS mutational analysis is affected not only by the input DNA quantity but also by several other factors, including the specimen type, the DNA quality, and the tumor cellularity. Here, we review tissue requirements for solid tumor NGS based mutational analysis, including procedure types, tissue types, tumor volume and fraction, decalcification, and treatment effects.

## 1. Introduction

The advent of next-generation sequencing (NGS) in routine clinical molecular diagnostics is facilitating alignment of cancer patients with appropriate targeted therapies through comprehensive mutation profiling [1,2,3,4,5,6,7,8,9,10,11,12,13,14,15]. Molecular oncology testing, especially of solid tumors is challenging because formalin fixation of surgical specimens compromises DNA integrity through chemical crosslinking of proteins and nucleic acids [16,17] and the yield of nucleic acid is often low from limited tissue specimens obtained through procedures such as fine-needle aspiration (FNA) and core-needle biopsy (CNB). These factors play a key role in selecting target enrichment method and NGS platform for mutation testing in solid tumors.

**Figure 1:**
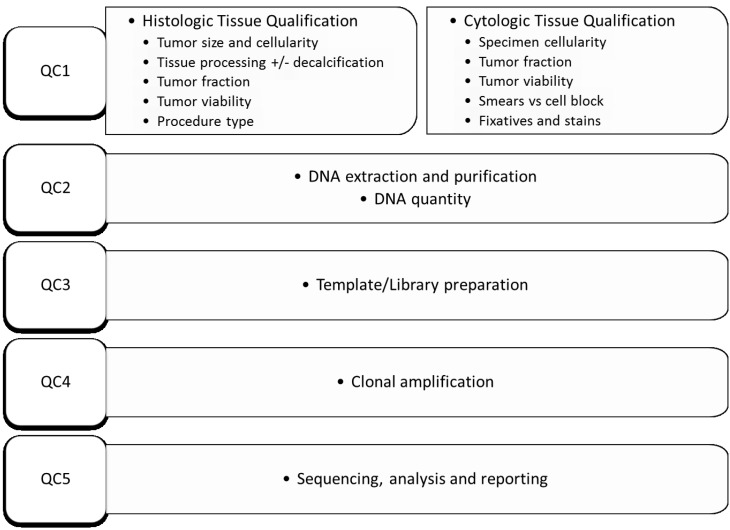
Illustration of sequential quality monitoring checkpoints (QC) for successful next-generation sequencing analysis based mutation analysis. The first checkpoint is pre-analytical tissue qualification; the important factors in tissue qualification are summarized here. Other checkpoints include DNA quantity, library preparation, clonal amplification, and sequencing and data analysis.

The two main types of NGS platforms used in molecular oncology clinical laboratories, the Ion Torrent PGM (Thermo Scientific) and MiSeq (Illumina) use multiplex PCR-based and/or hybridization capture–based target enrichment technology to generate the DNA library [1,2,8] (Table 1). Both have sequential quality control checkpoints at critical processing steps to ensure high-quality data is generated for the final analysis. The quality monitoring checkpoints include pre-analytical tissue qualification, DNA quantification, library preparation, clonal amplification, sequencing, and post-analytical data analysis [18] (Figure 1).

The amount of input DNA required for NGS depends on the platform, gene panel size and target enrichment method. For instance, testing for hotspots in 50 genes using the multiplex PCR based Ion AmpliSeq Cancer Hotspot Panel (Life Technologies) on the Ion Torrent PGM requires 10 ng of input DNA [2] whereas 250 ng is recommended for the similar hotspot TruSeq panel using MiSeq technology (Table 1). The sequencing error rate is slightly higher for the pH based Ion Torrent semiconductor detection technology (AQ20, 1 misaligned base per 100 bases) than for the fluorescence-based detection systems used in MiSeq (Q30, 1 incorrect call per 1000 bases) [19,20]; however, the low DNA input requirements of the Ion Torrent technology allows for testing of extremely small tumor samples, including FNA and CNB specimens of deep-seated organs [3].

Analyzing RNA sequences from clinical specimens also poses challenges including, potential degradation of RNA by RNases, RNA fragmentation from FFPE tissue, and low quantitative yield from small biopsy specimens. Despite these challenges, targeted RNA-Seq, such as anchored multiplex PCR on clinical FFPE samples has been successfully performed and reported [21]. In contrast to a DNA-based assay like NGS mutational analysis, where DNA quantity is critical for success, the success of RNA-Seq depends on the quality of RNA as determined by qPCR using internal housekeeping genes.

**Table 1:** Common commercial targeted and comprehensive sequencing panels for solid tumors.

Platform/panel parameters	Ion AmpliSeq^TM^ Cancer Hotspot Panel v2	Ion AmpliSeq^TM^ Comprehensive Cancer Panel	TruSeq Amplicon Cancer Panel	TruSight^TM^ Tumor Sequencing Panel
NGS platform, Manufacturer	Ion Torrent, Life Technologies	Ion Proton, Life Technologies	MiSeq or NextSeq, Illumina	MiSeq or NextSeq, Illumina
Sample type	FFPE, cytology	FFPE, cytology	FFPE, cytology *	FFPE, cytology
DNA input	10 ng	40 ng	250 ng	30–300 ng
Genes in the panel	50	409	48	26
Library preparation	Multiplexed PCR	Multiplexed PCR	Multiplexed probe-based capture	Multiplexed probe-based capture
Amplicon size	111–187 bp	125–175 bp	170–190 bp	165–195 bp
Number of amplicons	207	~16,000	212	174
Sequencing technique	Semiconductor sequencing	Semiconductor sequencing	Fluorescence based sequencing-by-synthesis	Fluorescence based sequencing-by-synthesis
Sequencing quality cutoff	AQ 20	AQ 20	Q30	Q30

* only specimens with high cellularity meeting minimum DNA threshold.

Pre-analytical tissue selection plays a key role in the subsequent success of NGS mutational analysis. To ensure selection of appropriate tissue, most institutions have designated surgical pathologists and/or cytopathologists to select appropriate surgical tissue sections or cytologic preparations that best meet the requirements of the NGS platform. The pathologists circle tumor-rich areas of the tissue sections on the slides and quantify the tumor fraction to meet adequacy criteria for NGS mutational analysis. In the case of patients for whom multiple specimens or tumor blocks and/or cytologic specimens are available for biomarker testing, the most recent specimen from the metastasis or recurrence is usually recommended for testing. In this review, we discuss the pre-analytical tissue factors that are associated with the NGS performance, focusing on DNA-based mutational analysis (Table 1 and Table 2). Since NGS mutational analysis on solid tumors is a relatively new area for clinical laboratories; currently very few guidelines are available and they mainly focus on analytical wet bench processes, bioinformatics and reporting [22]. This review will provide the guideline regarding pre-analytical tissue factors for solid tumor NGS mutational analysis.

**Table 2:** Pre-analytical factors associated with success of next-generation sequencing (NGS) mutational analysis of solid tumor specimens.

Associated Factors	Comments
Tissue processing and storage	Formalin-fixed paraffin-embedded tissue (FFPE)
	Standard for histologic evaluation and long term storage (in years) DNA fragmented due to formalin fixation Tumor fraction in unstained FFPE section can be approximated from mirror H&E section
	Frozen tissue
	Intact DNA suitable for molecular testing Long term storage at −80 °C Presence of tumor in tissue and tumor fraction cannot be accurately evaluated
Tumor size and cellularity	Tumor size
	Large and cellular lesions compatible with most NGS platforms Extremely small lesions may work on NGS platforms requiring low DNA input
	Tumor cellularity
	High cellularity and larger tumors compatible with most NGS platforms Low-cellularity tumors require more unstained tumor sections for DNA extraction Tumor cellularity depends on nature of tumor
Type of procedure	Surgical resection and excision
	Usually associated with large tumor size and high DNA yield
	Superficial biopsy
	Variable size and cellularity
	Imaging-guided biopsy
	Usually associated with small or extremely small tumor and low DNA yield
Tumor type and tumor site	Tumor type
	Nature of tumor (solid *vs* cystic *vs* sclerotic *vs* mucinous) Primary and metastatic bone tumors (decalcification procedures)
	Tumor site
	Diagnostic material accessibility (superficial *vs* deep *vs* imaging guided) Bone (decalcification procedures)
Tumor fraction	Tumor fraction determinants
	Tumor type, primary *vs* metastasis, and associated stromal and inflammatory cells
	Tumor fraction requirement for NGS
	Depends on technical sensitivity of NGS platform
DNA yield and DNA input	DNA yield
	Depends on selected tumor size and cellularity
	DNA input
	DNA input at manufacturer recommendation associated with high NGS success; may be able to use less DNA
Tumor viability	Viable tumor
	Best for histologic evaluation and PCR-based sequencing
	Nonviable tumor from apoptosis
	May be compatible with some PCR-based sequencing using short targeted sequences
	Nonviable or necrotic tumor from autolysis
	Incompatible with PCR based sequencing
Decalcification of bone	Strong acid–based decalcification
	Rapid Good for histologic evaluation Not suitable for PCR-based sequencing
	Weak acid–based decalcification
	Slow Very good for histologic evaluation Compatible with PCR-based sequencing
	Chelating agent–based decalcification
	Extremely slow Best for histologic evaluation Good for PCR-based sequencing
Other factors	Blood
	Heme in red blood cells may inhibit PCR reaction
	Mucin
	May lower tumor cellularity

NGS, next-generation sequencing; H&E, hematoxylin and eosin.

## 2. Histologic Specimens

### 2.1. Tissue Processing and Storage

Tissues procured *via* biopsy and surgery are routinely processed through formalin fixation after gross examination and tissue sectioning. The selected tissue sections are embedded in paraffin blocks, which are then used to prepare thin, 4-5-micron sections for hematoxylin and eosin (H&E) stain and ancillary studies such as special staining, immunohistochemistry staining, *in situ* hybridization, and molecular testing. The H&E staining and ancillary studies of the formalin-fixed paraffin-embedded tissue (FFPE) sections are reviewed by pathologists to provide the final pathological diagnosis. Pathology reporting of malignant neoplasms includes tumor grade, stage, size, biomarker status, nodal involvement, and response to neoadjuvant therapy if indicated, and tumor block for future biomarker testing at The University of MD Anderson Cancer Center (MD Anderson).

Formalin, the standard tissue fixative in most anatomical pathology laboratories, is an aqueous 37% solution of formaldehyde, commonly used as a neutral buffered formalin solution at 10% concentration (approximately 4% formaldehyde). It preserves tissue in relatively close approximation to its *in vivo* morphology by fixing both cytoskeletal and soluble proteins [23] Thus, formalin fixation provides excellent preservation of morphology. However, reversible cross links form between proteins and nucleic acids, in addition to random breaks that occur, resulting in highly fragmented nuclei acids [17]. Furthermore, FFPE derived nucleic acid is only compatible with PCR based assays designed for short target sequences.

On the other hand, FFPE tissue is the standard substrate for most clinical molecular laboratory testing, since it represents the majority of tissue sample processed in a clinical pathology laboratory. The FFPE tissue blocks can be stored at room temperatures for years at a low cost and can be used for both histologic examination as well as for selected ancillary studies. Using sequential cuts from the FFPE tissue block, the tumor fraction in unstained sections can be estimated using a representative mirror H&E stained section. In contrast, the collection and storage of frozen tissue is relatively costly and resource intensive. Although frozen tissue provides unfragmented and high quality DNA, the presence of tumor and tumor fraction cannot always be accurately evaluated.

### 2.2. Tumor Size and Cellularity

The size of the selected tumor-rich areas and number of viable tumor cells within those areas determine the DNA yield, a critical quality control step for NGS. The expected average DNA yield from a single nucleated cell is approximately 6 pg. To obtain the 10 ng recommended DNA for the Ion AmpliSeq Cancer Hotspot Panel, approximately 2000 whole cells are required. In a typical large sample of excised tumor, a single unstained FFPE section with at least 60–100 mm^2^ of selected tumor-rich tissue would be required to provide the 2000 cells necessary to yield adequate DNA for NGS mutational analysis. In contrast, extremely small tumor sections with <10 mm^2^ of selected tumor-rich tissue often fall short of providing adequate DNA, even if tissue is extracted from multiple (10 to 20) unstained FFPE sections [24].

In our experience, as expected, the NGS failure rate due to inadequate DNA quantity is usually much higher in tumors from which the selected tumor tissue sections are extremely small than in tumors from which the selected sections are larger [24]. Increasing the tissue size by selecting a larger area may increase the DNA yield, but this increase often is at the expense of decreasing the tumor fraction by including more non-tumor components, such as stromal cells. When the estimated tumor fraction is less than the analytical sensitivity of the NGS platform (approximately 5%), the risk of false-negative calls is high and the unequivocal determination of low-frequency variant calls is difficult.

Hypocellular tumors require that larger areas be selected and thus greater numbers of unstained sections are needed to ensure adequacy of tumor extraction and cellularity for DNA purification. Common hypocellular specimens include mucinous tumors with scant tumor cells floating in large pools of mucin and treated tumors with residual tumor cells in hyalinized fibrotic stroma (Table 2).

### 2.3. Type of Procedure

Solid tumor specimens tested by NGS are from surgical resections, excisions, or CNB or FNA procedures. The surgical resection and excision specimens generally provide large tumor sections and yield more DNA than the smaller CNB and FNA specimens and are associated with higher NGS success rates [24]. However, a large fraction of diagnostic samples are obtained by minimally invasive procedures such as FNA and CNB. Not only do these samples tend to be smaller and yield less DNA to begin with, but often they get smaller with subsequent recutting of deeper sections used for molecular biomarker testing. Consequently, when compared side by side, the NGS success rate is lower in small samples (CNB and FNA specimens) than in larger samples (resections and excisions), when the manufacturer-recommended DNA input amount is strictly followed [24]. However, if the input DNA threshold is lowered, a subset of cases with high-quality DNA but low DNA concentration can be sequenced successfully on the Ion Torrent platform, allowing uniform NGS success rates across all procedure types (resection, excision, CNB, and FNA) [24,25].

### 2.4. Tumor Type and Tumor Site

Deep-seated tumors require image-guided FNA/biopsy by an interventional radiologist. They can often pose difficulties in terms of accessibility of lesion, acquisition of adequate material, and risk of bleeding and other complications [26]. With recent advances in imaging technology, our ability to detect smaller lesions is improving. Often the interventional radiologist has to use a small caliber (18 to 20 gauge) needle to sample these sub-centimeter lesions. The tumor tissue obtained by these smaller needles is generally scant and thus the DNA yield is low. In small and/or technically challenging lesions, the interventional radiologist often will forego the CNB and perform an FNA with a smaller (20 to 23 gauge) needle to minimize potential complications associated with the procedure [26]. This underscores the need for novel molecular techniques such as NGS that can provide highly multiplexed and massively parallel sequencing for multiple genes using very small quantities of input DNA. A study performed at our center that reviewed various types of solid tumors sampled by various techniques (resection, excision, CNB, and FNA) showed that NGS success rates were similar across all tumor sites and types after adjusting for tumor size and absence of decalcification [24].

### 2.5. Tumor Fraction

The tumor fraction requirement for NGS is dependent on the analytical sensitivity of the platform. The lowest limit of detection (*i.e.*, analytical sensitivity) for low-frequency variants is approximately 5%–10% in deep sequencing coverage of 250 to 500× [2,5,8,27,28]. Since a majority of tumors are heterozygous, and somatic mutations usually affect one of two alleles in oncogenes, the minimum tumor fraction within selected areas is 10%–20%. Because ultra-deep sequencing can detect variants at <1% frequency, the minimum tumor fraction can drop below 10% [29]. In specimens matched for tumor size with tumor fractions >20%, the NGS success rates on the Ion Torrent platform are similar across various tumor fractions [24,30].

### 2.6. DNA Yield and DNA Input

The DNA input is a critical quality control step to achieve the highest success rate for NGS mutational analysis of solid tumors. However, it should be noted that strict adherence to the manufacturer-recommended DNA input, such as 10 ng for Ion AmpliSeq Cancer Hotspot panel can exclude a small subset of samples with borderline DNA yield that is of sufficiently high quality but falls short of the recommended DNA input threshold. A validation study at MD Anderson showed that NGS can be performed successfully in solid tumor samples with DNA input less than the manufacturer’s recommendation, improving the overall NGS success rate [24,25]. This allows a larger number of extremely small tumor samples, such as those obtained from minimally invasive procedures like FNAs and CNBs, to be analyzed successfully with NGS.

### 2.7. Tumor Viability

Viable tumor tissue is preferred for molecular testing [31]. However, tumor necrosis is not infrequently observed in tumors such as colorectal adenocarcinoma, squamous cell carcinoma, and glioblastoma, as well as in tumors that have undergone neoadjuvant therapy. Cell death can be caused by either apoptosis or necrosis. Necrosis typically leads to autolysis of cellular components, including karyolysis, which is incompatible with PCR-based sequencing tests. In contrast, apoptosis, also known as programmed cell death, is associated with nuclear and DNA fragmentation, which is presumed to be compatible with PCR-based sequencing tests if the target sequences are short. Many chemotherapy drugs such as antimetabolites and alkylating agents exert their antitumor effects through apoptosis. Therefore, non-viable tumor in post-treatment samples, which may appear dead morphologically, can yield fragmented DNA that can still be used for molecular testing. A study on areas of necrosis in colorectal adenocarcinomas generated results comparable to those of viable tumor on NGS mutational analysis by the Ion AmpliSeq Cancer Hotspot Panel [32]. However, the findings are preliminary and further studies are needed to evaluate the effect of tumor necrosis, both in treated tumors and in other necrotic tumor types.

### 2.8. Decalcification of Bone Specimens

Mineralized bone specimens are hard and brittle after routine histologic processing, making production of optimal sections for histologic evaluation and ancillary studies extremely difficult [33]. A decalcification process removes calcium ions from bone for routine histologic processing and evaluation. Decalcifying reagents typically contain a strong acid (nitric acid or hydrochloric acid), a weak acid (picric, acetic, or formic acid), or a chelating agent (EDTA) [34]. Adequate tissue fixation is required prior to decalcification to protect the cellular and stromal components of bone and attached soft tissue from damage.

Strong acid–based decalcification procedures use acids of extremely low pH to achieve the most rapid removal of calcium ion from bone [35,36]; however, prolonged fixation in strong acid rapidly macerates the tissue and prevents nuclear staining (basic staining). In addition, strong acids tend to degrade DNA, making the decalcified bone sections suboptimal for PCR-based molecular testing [34].

Weak acids, although slower than the strong acid agents in the decalcifying process, are much gentler in action and less likely to interfere with nuclear staining [33]. Thus, formic acid–based decalcification is compatible with histologic evaluation, immunohistochemical studies, and PCR-based sequencing analysis. The slow decalcification induced by the formic acid-based decalcification protocol, for example, allows repeated monitoring of the specimen by radiographic methods to evaluate decalcification status, which permits optimization of results. The process is halted with a neutralizing buffer once decalcification is noted in the specimen, grossly or radiographically. A study at our institution showed the success rate of NGS performed on the Ion Torrent platform reached more than 70% in bony specimens processed with a formic acid–based decalcification procedure; only slightly lower than that achieved in solid tumors from non-bony sites without decalcification [24].

Chelating agents work very slowly, and the decalcifying process with these agents may take weeks [34]. EDTA captures calcium ions from apatite crystal to slowly reduce its size. Although the EDTA decalcifying process is very gentle and preserves morphology and tissue features for immunohistochemistry, *in situ* hybridization, and PCR-based molecular testing [34], the extremely slow process limits its use in clinical laboratories that must respond to needs for time-sensitive diagnostic information critical for patient management.

Therefore, bony samples that have undergone strong acid-based decalcification are compatible with histologic evaluation but are not suitable for PCR-based sequencing procedures, whereas samples that undergo formic acid- or EDTA-based decalcification procedures take longer to process but are suitable for both morphologic analysis and PCR-based sequencing, including NGS. Ultimately the choice of decalcifying agent will depend on the specific laboratory and balancing the priorities for turnaround time, high-quality histologic sections, and reliable molecular results by the laboratory.

### 2.9. Other Factors: Blood and Mucin

Although anucleated blood cells such as red blood cells (RBCs) do not reduce the tumor fraction, heme in RBCs can act as an inhibitor in the PCR reaction [37]. Therefore, special care is needed during selection of tumor areas to enrich the tumor fraction while minimizing entrapped RBCs in bloody specimens. Mucin and hyalinized stroma in tumor samples do not affect the PCR reaction per se; however, large quantities of these in the tissue section can reduce the overall tumor cellularity and dilute the sample. Therefore, appropriate numbers of unstained tumor sections are usually required to ensure sufficient tumor cells for DNA extraction.

## 3. Cytology Specimens

As previously mentioned, it is becoming increasingly common to perform NGS mutational analysis on cytology specimens. FNA procedures, which are minimally invasive and generally well tolerated by the patient, can provide adequate material for diagnosis as well as ancillary studies, including NGS mutational analysis [3,25,38,39]. Cytologic specimens offer a number of advantages in terms of molecular testing. At many institutions, rapid on-site evaluation (ROSE) guides the interventional radiologist in acquiring an adequate specimen, which can be assessed immediately for diagnosis as well as ancillary studies. Direct smears, cytospin preparations, and liquid-based cytologic preparations (LBC) are not formalin-fixed and provide whole cells with whole nuclei, unlike FFPE sections which are typically 4 or 5 microns in thickness, and therefore yield higher quality/quantity DNA [38]. FNAs typically offer wider sampling of the lesion since, unlike CNB specimens, the aspiration is not unidirectional. In addition, FNA specimens usually contain fewer background benign cells (e.g., stromal cells) and therefore have a higher tumor fraction than other types of specimens [38]. Finally, cytologic bone samples are not decalcified, and therefore DNA quality is not compromised by acid or harsh fixatives [38,40]. However, cytology specimens are historically underutilized for molecular testing [41], primarily because most molecular testing requests are directed reflexively to the histology specimens, and cytology specimens are typically tested only when there is no concurrent core biopsy or the core biopsy is depleted/insufficient for testing [25]. The other issue that complicates testing of cytology specimens is that molecular laboratories are not always validated to test all the different types of cytologic specimen preparations (direct smears, cytospin preparations, LBC, and FFPE cell blocks), and most laboratories prefer to treat all specimens as they do traditional FFPE histology blocks [40]. Some of the pre-analytical factors affecting NGS mutational analysis in cytology specimens are discussed below.

### 3.1. Specimen Cellularity

In cytology, as in histology specimens, the overall cellularity plays an important role in NGS mutational analysis. For routine NGS mutational analysis on an Ion Torrent PGM system requiring 10 ng input DNA for a hotspot panel, as already stated, the goal is for a specimen with a total of at least 2000 cells. The specimen cellularity for FNAs depends largely on the quality of the procedure (*i.e.*, skill and expertise of the proceduralist) and the nature of the lesion (*i.e.*, size, location and cellular cohesion of the tumor). Small sub-centimeter lesions as well as deep-seated tumors that are technically challenging to aspirate typically have a lower cellular yield. In these cases, the cellularity may be a function of the skill and experience of the aspirator. In addition, the nature of the tumor may also play a role in the cellularity. For example, sclerotic or cystic tumors tend to be paucicellular, while tumors with largely discohesive cells, like melanoma or neuroendocrine tumors, usually yield highly cellular aspirates (Table 3).

### 3.2. Type of Preparation

While specimen cellularity may depend largely on the FNA procedure, the cytologic preparation also plays an important role in the choice of a substrate for NGS testing. Cytology offers a multitude of preparations for NGS mutational analysis, including direct smears and cytospins, FFPE cell blocks, and LBC. Cell block preparations, although easiest to use and validate, in our experience and others, often fall short of providing adequate material [38,42,43]. The biggest disadvantage of cell block preparations is the inability to assess them immediately for adequacy. While implementing dedicated FNA passes for the needle rinse may enhance cellularity for the cell block, it still does not guarantee an adequate sample for ancillary studies. LBC preparations share the same disadvantages in being not immediately assessable and having variable neoplastic cellularity; however, the overall ease of specimen collection and transportation, combined with an optimally preserved sample that largely eliminates background blood and debris, make LBC an attractive sample preparation for NGS testing [44]. Most institutions that perform NGS testing from LBC preparations employ some form of pre-analytical measure through visualization of neoplastic cells in the LBC preparation to avoid false-negative calls [45,46,47]. In contrast, direct smears provide the only source of reliable testing material in cases with limited tissue and where the cell block is inadequate for testing. DNA obtained from direct smears do not have formalin fixation artifacts [17,25,40] and provide whole cells with intact nuclei (Table 3). However, utilizing direct smears for NGS mutational analysis requires sacrificing the slide, which may be a medicolegal issue in certain states and institutions, as well as additional in-house validation for performing the NGS assay.

**Table 3:** Pre-analytical factors in cytologic specimens associated with success of NGS mutational analysis.

Associated Factors	Comments
Specimen cellularity	Depends on multiple factors, including
	Size and location of tumor Skill and experience of aspirator Nature of tumor (e.g., solid *vs* cystic *vs* sclerotic)Type of tumor (discohesive cells such as melanoma, neuroendocrine carcinoma usually have higher yield)
Type of preparation	Direct smears
	Immediate assessment 3-dimensional clusters with whole nuclei Usually better quality DNA than cell blocks No formalin fixation artifact Requires in-house validation Requires sacrificing of the slide
	Cell blocks
	Variable cellularity; no immediate assessment Formalin-fixed cells 4- to 5-micron sections with partial nuclei Easy to validateLiquid-based preparations
	Liquid-based preparations
	Lack of immediate assessment Ease of specimen collection and transportation Optimal preservation of nucleic acids Requires in-house validation
Type of fixative and stains	Direct smears
	Air-dried methanol fixed, Diff-Quik stained Ethanol fixed, Papanicolaou (Pap) stained
Type of glass slide	Non-frosted *vs* positively charged *vs* fully frosted slides
Tumor fraction	Tumor fraction determinants
	Tumor type, primary *vs* metastasis Specimen type, fluids and EBUS-guided lymph node FNAs often have low tumor fraction
	Tumor fraction requirement for NGS
	Depends on technical sensitivity of NGS platform
DNA yield	Depends on the overall cellularity of sample
	Samples with high tumor fraction but scant cellularity may not have adequate DNA yield for PCR amplification
Input DNA	High-quality/low-DNA-concentration samples can benefit from lowering the threshold of input DNA

NGS, next-generation sequencing; EBUS, endobronchial ultrasound; FNA, fine needle aspiration.

### 3.3. Type of Fixative and Stains

Studies by several groups have shown that both methanol-fixed Diff-Quik-stained smears as well as ethanol-fixed Papanicolaou (Pap)-stained smears can be used for molecular testing [44,48,49,50]. However, interlaboratory variation in fixation and staining techniques may play a role in DNA yield and molecular testing success. In our experience there is no significant difference in NGS mutational analysis findings between different cytologic fixatives and stains [3].

### 3.4. Type of Slide

A variety of glass slides are commercially available for preparation of cytologic direct smears and cytospins. These include the standard non-frosted or frosted tip slides that do not have any specialized coating; the fully frosted slides that have a rough surface to enhance cellular adhesion; the positively charged slides that electrostatically enhance cellular adhesion; and coated slides that use silane or lysine allowing covalent bonding of cells to the slide surface. It is reasonable to assume the type of slide used for specimen preparation plays a role in the cellularity seen on the smears. In our experience fully frosted slides show enhanced cellular retention, with lower cell loss during fixation and processing, than the frosted tip or positively charged slides. However, glass slides that enhance cellular adherence, can also pose difficulties during tissue extraction. A recent study by our group showed that the frosted tip slides yield higher DNA quantities than the fully frosted slides, presumably due to the difficulty in dislodging cells from the rough surface of the fully frosted slides [25]. Therefore, the choice of glass slide type can be an important factor in the success of NGS (Table 3).

### 3.5. Tumor Fraction

As mentioned earlier, the tumor fraction requirement for NGS is dependent on the analytical sensitivity of the platform which is approximately 5%–10% in deep sequencing coverage of 250 to 500×. Assuming most tumors are heterozygous, the minimum tumor fraction within tumor enriched areas needs to be approximately 10%–20%. In cytology, some specimens have an intrinsically low tumor fraction due to the abundant benign cells in the background. Examples would include pleural and peritoneal fluids with contaminating mesothelial cells and histiocytes and samples obtained through endobronchial ultrasound guided (EBUS) FNA with background lymphocytes and bronchial cells (Table 3).

Cytology specimens like histology specimens are enriched for tumor by identifying areas on the slide with a higher tumor fraction for tissue extraction. Cell block sections are treated the same as histology specimens: the selected tumor-rich area of the hematoxylin and eosin (H&E)-stained section is circled and the tumor fraction estimated by the cytopathologist. Unstained sections are then matched up with the H&E slide and the corresponding areas are manually macro/microdissected using a scalpel blade. For direct smear slides, the selected tumor-rich area is circled on the slide and then the back of the slide is etched with a diamond-tip pen in the area corresponding to the circled tumor-rich area. The slide is then dipped in xylene to remove the coverslip. The process of removing the coverslip from archival slides does not affect the DNA yield [40,48]. Alternatively, representative slides can be triaged at the time of rapid on-site evaluation and left uncoverslipped for DNA extraction and molecular analysis. The circled/etched areas on the slides can then be macro/microdissected using a scalpel blade.

### 3.6. DNA Yield and DNA Input

As previously mentioned, adhering to the manufacturer-recommended DNA input can exclude a small subset of samples with borderline DNA yield that falls short of the recommended DNA input threshold but is of sufficiently high quality for NGS success. A study from MD Anderson showed that the overall NGS success rates were significantly improved in cytology samples using DNA input less than the manufacturer’s recommendation [25].

## 4. Summary

The success of NGS mutational analysis in solid tumors is affected not only by the input DNA quantity but also by a constellation of factors including the DNA quality, the tumor fraction, and the nucleic acid extraction method. To meet the nucleic acid requirement for the testing platform, dedicated pathologists select viable and cellular tumor sections/smears to optimize the tumor-to-stroma ratio and to minimize the admixed inflammatory cells, blood, mucin, and necrotic tumor cells. Sections of decalcified bone may be chosen with caution if a weak acid- or EDTA-based decalcification protocol has been properly administered. Pre-analytical qualification of tissues from solid tumor specimens would ensure a high rate of success in subsequent NGS steps to achieve overall high-quality NGS mutational analysis results.
